# Supplementary material for: Resident and recruited macrophages differentially contribute to cardiac healing after myocardial ischemia
Source: eLife. 2024 May 22;12:RP89377. doi: 10.7554/eLife.89377 (PMC11111219; doi:10.7554/eLife.89377)
Supplement: Supplementary file 1. [file elife-89377-supp1.docx]

| Antibody | Dilution | Clone | Fluorescence | Company | Catalog number |
| --- | --- | --- | --- | --- | --- |
| Flow Cytometry | | | | | |
| CD115 | 1:100 | AFS98 | BV421 | Biolegend | 135513 |
| CD115 | 1:100 | AFS98 | APC | Biolegend | 135509 |
| CD11b | 1:100 | M1/70 | PE-Cy7 | BD Biosciences | 552850 |
| CD11b | 1:100 |  | APC-Cy7 |  |  |
| CD11c | 1:100 | \| N418 \| \| --- \| | PE | Biolegend | 117308 |
| CD11c | 1:100 |  | Fitc |  |  |
| CD16/CD32 (FcγRIII/FcγRII) | 1:100 | 2.4G2 | uncoupled | BD Biosciences | 553142 |
| CD19 | 1:200 |  |  |  |  |
| CD64 | 1:100 | X54-5/7.1 | APC | Biolegend | 139306 |
| CD45 | 1:100 |  | BUV395 |  |  |
| CD45 | 1:100 | 30-F11 | PerCP | Biolegend | 103130 |
| CD45.1 | 1:100 | A20 | Fitc | BD Biosciences | 110706 |
| CD45.2 | 1:100 | 104 | APC-Cy7 | BD Biosciences | 560694 |
| F4/80 | 1:100 | BM8 | BV421 | Biolegend | 123131 |
| Gr-1 | 1:100 | RB6-8C5 | bio | eBio | 13-5931-82 |
| Gr-1 | 1:100 |  |  |  |  |
| Ly6G | 1:100 | 1A8 | BV605 | Biolegend | 127639 |
| NK1.1 | 1:100 | PK136 | PE | Biolegend | 108708 |
| NK1.1 | 1:100 |  | Fitc |  |  |
| TCR-ß | 1:100 | 1B3.3 | PE | Biolegend | 109208 |
| TCR-ß | 1:100 |  | Fitc |  |  |
| Ter119 | 1:100 | TER-119 | PE | Biolegend | 116208 |
| Ter119 | 1:100 |  | Fitc |  |  |
| I-A/I-E Antibody | 1:100 | M5/114.15.2 | PE/Cy7 | Biolegend | 107630 |
| SYTOX™ Orange | 1:1000 |  | PE | Thermofisher | S11368 |
| CD45 MicroBeads, mouse | 1:100 | 30F11.1 |  | Miltenyi Biotec | 130-052-301 |
| Immunohistology | | | | | |
| anti-GFP | 1:100 | Polyclonal, Rabbit | uncoupled | Invitrogen | A-11122 |
| CD68 | 1:200 | FA11 | uncoupled | BioRad | MCA1957 |
| Wheat Germ Agglutinin | 1:100 |  | Alexa Fluor 350 | ThermoFisher Scientific | W11263 |
| AF488 goat anti-rabbit | 1:200 | Polyclonal | Alexa Fluor 488 | Invitrogen | A-11034 |
| AF 555 goat anti rat | 1:200 | Polyclonal | Alexa Fluor 555 | Invitrogen | A-21343 |

**Supplementary File 1. Antibody list.**
